# cRGD-Conjugated GdIO Nanoclusters for the Theranostics of Pancreatic Cancer through the Combination of T_1_–T_2_ Dual-Modal MRI and DTX Delivery

**DOI:** 10.3390/molecules28166134

**Published:** 2023-08-18

**Authors:** Shengchao Wang, Guiqiang Qi, Zhichen Zhang, Qiangqiang Yin, Na Li, Zhongtao Li, Guangyue Shi, Haifeng Hu, Liguo Hao

**Affiliations:** 1 Department of Molecular Imaging, School of Medical Technology, Qiqihar Medical University, Qiqihar 161006, China; wangsc1163@163.com (S.W.);; 2 Department of Imaging Medicine and Nuclear Medicine, School of Clinical Medicine, Jiamusi University, Jiamusi 154002, China; 3 Medical Imaging Center, The Second Affiliated Hospital of Qiqihar Medical University, Qiqihar 161000, China

**Keywords:** T_1_–T_2_, DTX, MRI, drug delivery, pancreatic cancer

## Abstract

Clinically, magnetic resonance imaging (MRI) often uses contrast agents (CAs) to improve image contrast, but single-signal MRI CAs are often susceptible to calcification, hemorrhage, and magnetic sensitivity. Herein, iron acetylacetone and gadolinium acetylacetone were used as raw materials to synthesize a T_1_–T_2_ dual-mode imaging gadolinium-doped iron oxide (GdIO) nanocluster. Moreover, to endow the nanoclusters with targeting properties and achieve antitumor effects, the cyclic Arg-Gly-Asp (cRGD) peptide and docetaxel (DTX) were attached to the nanocluster surface, and the efficacy of the decorated nanoclusters against pancreatic cancer was evaluated. The final synthesized material cRGD-GdIO-DTX actively targeted α_v_β_3_ on the surface of Panc-1 pancreatic cancer cells. Compared with conventional passive targeting, the enrichment of cRGD-GdIO-DTX in tumor tissues improved, and the diagnostic accuracy was significantly enhanced. Moreover, the acidic tumor microenvironment triggered the release of DTX from cRGD-GdIO-DTX, thus achieving tumor treatment. The inhibition of the proliferation of SW1990 and Panc-1 pancreatic cancer cells by cRGD-GdIO-DTX was much stronger than that by the untargeted GdIO-DTX and free DTX in vitro. In addition, in a human pancreatic cancer xenograft model, cRGD-GdIO-DTX considerably slowed tumor development and demonstrated excellent magnetic resonance enhancement. Our results suggest that cRGD-GdIO-DTX has potential applications for the precise diagnosis and efficient treatment of pancreatic cancer.

## 1. Introduction

Pancreatic cancer is the digestive tract malignancy with the worst prognosis, with a five-year survival rate of 5% to 9% [1]. Pancreatic cancer fatalities and newly diagnosed cases both increased in 2020, reaching 495,773 cases and 466,003 deaths worldwide, respectively [2], which has made this disease a severe threat to human health. The key to overcoming cancer is precise diagnosis and early treatment [3,4]. Due to its noninvasive nature, lack of ionizing radiation, and excellent resolution for soft tissue, magnetic resonance imaging (MRI) has garnered much interest in recent years for clinical diagnosis, particularly for malignant tumors [5,6,7]. It would therefore be advantageous to utilize contrast agents (CAs), which are frequently used to enhance the MRI signals in diseased tissue to distinguish it from normal tissue by finding pathological abnormalities [8,9]. Currently, the most widely used CAs in the clinic are the T_1_ CAs Magnevist [10] and the T_2_ CAs Feridex [11]. However, the majority of clinical MRI CAs are nontargeting substances and extremely prone to excretion out of the body and passive and nonspecific distribution into the tissue’s interstitial space, which might occasionally lead to unwanted MRI contrast enhancement [12]. Therefore, it is possible that new CAs may be developed in the future, which might lead to more precise accumulation in diseased tissue and aid in the collection of more precise diagnostic data [13]. Arg-Gly-Asp (RGD) is a site at which integrin α_v_β_3_ recognizes its ligand, and this sequence can specifically recognize overexpressed α_v_β_3_ in pancreatic cancer cells [14]. Hence, utilization of this sequence could improve tumor targeting. Therefore, the targeted cRGD peptide would be good candidates for α_v_β_3_-overexpressing tumors, including pancreatic cancer.

As previously mentioned [15,16], T_1_ CAs mostly reduce the spin-lattice relaxation time of protons, whereas T_2_ CAs primarily speed up the attenuation of the spin–spin relaxation time of water molecules. However, in vivo single-mode T_1_ or T_2_ magnetic resonance imaging is very prone to interference from calcification, hemorrhage, or metal deposits, which greatly restrict their ability to accurately diagnose diseased tissue [17]. Due to the benefits of integrating T_1_ and T_2_ CAs, the creation of T_1_–T_2_ dual-mode MRI CAs has recently received much attention [18]. It has been demonstrated that T_1_–T_2_ dual-mode MRI CAs may considerably increase the detection accuracy by providing complementary information about a lesion for self-confirmation and offering error-free MR images [19,20].

Docetaxel (DTX) is a compound synthesized by the structural modification of a compound extracted from yew berries and needles, which is the most promising drug in from yew trees, and has received FDA approval in the United States for the treatment of solid tumors such as cervical cancer, breast cancer and a range of other cancerous tumors [21]. Similar to its counterpart paclitaxel, DTX interrupts cell division by stabilizing the microtubule structure to inhibit the development of tumor cells and promote apoptosis [22]. DTX has effective anticancer properties, but its hydrophobicity has restricted its practical use for intravenous administration [23]. Tween 80 is typically used to increase the solubility of DTX; however, it has the potential to induce significant neurotoxicity and hypersensitivity reactions [24]. Therefore, there is an urgent need to develop a new mode of DTX delivery with high water solubility, good selective drug distribution, and few side effects. In addition to helping anticancer medications reach their target tissues, nanocarriers can reduce side effects, enhance the antitumor benefits and improve biocompatibility with long-lasting stability [25,26,27].

In this study, we synthesized a novel multifunctional gadolinium-doped iron oxide nanocluster (GdIO) that can be used for T_1_–T_2_ imaging with RGD receptor specificity for magnetic resonance imaging-targeted DTX delivery. The results showed that cRGD-GdIO-DTX can bind specifically to the α_v_β_3_ receptor on the surface of pancreatic cancer cells, degrade rapidly under the reduced pH conditions of the tumor microenvironment, and release the loaded DTX. In a human pancreatic cancer xenograft model, systemic delivery of cRGD-GdIO-DTX dramatically restricted the development of tumors and showed excellent MR enhancement.

## 2. Results and Discussion

### 2.1. Synthesis and Characterization

**Scheme 1:**
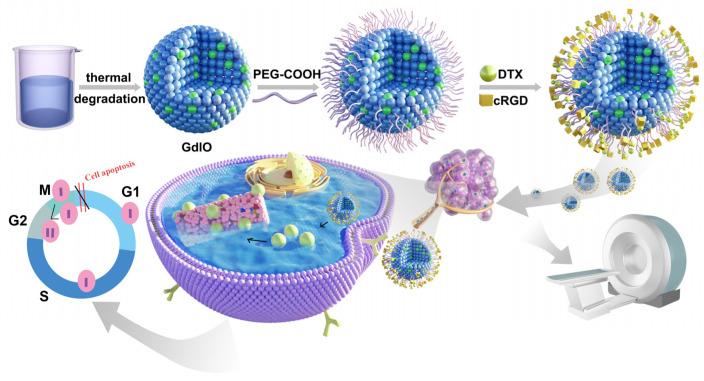
cRGD-GdIO-DTX synthesis process and the mechanism of diagnosis and treatment.

**Figure 1:**
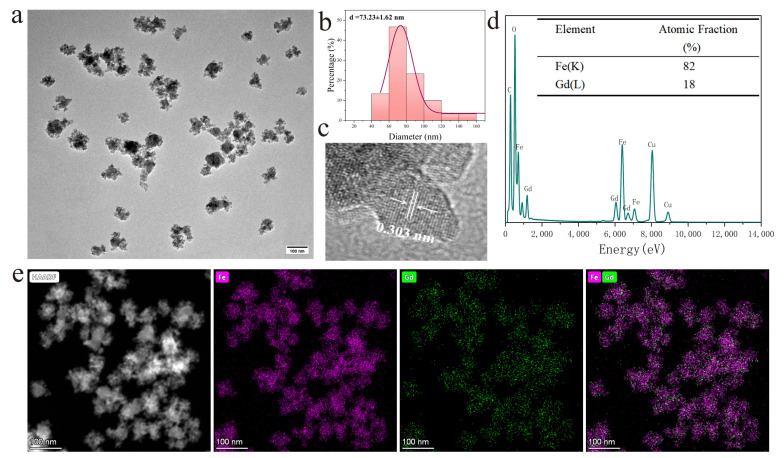
(**a**) TEM and (**c**) HRTEM images of GdIO; (**b**) Size distribution histograms; (**d**) EDS spectrum of GdIO; inset: table showing the quantification of the respective element. (**e**) EDS elemental mapping of GdIO.

Nanomaterials can effectively integrate diagnostic and therapeutic processes. In this study, we synthesized a hybrid nanocarrier for magnetic resonance imaging and drug delivery. Scheme 1 illustrates the synthesis of cRGD-GdIO-DTX. First, GdIO nanoclusters were synthesized. Subsequently, the surface of the nanoclusters was modified using PEG_600_ diacid (COOH-PEG_600_-COOH). Next, cRGD and DTX were chemically linked to the nanocluster surface. The transmission electron microscopy (TEM) images in Figure 1a, b show that the nanoclusters were evenly dispersed in water without obvious aggregation with an average size of 73.23 ± 1.62 nm. As measured by high-resolution transmission electron microscopy (HRTEM), the separation of two neighboring planes of the nanocluster was 0.303 nm, corresponding to the (220) crystal plane of cubic magnetic Fe_3_O_4_, indicating that the main crystal structure of the nanocluster is Fe_3_O_4_. However, compared with the standard (220) crystal plane of 0.296 nm, the crystal plane spacing was slightly larger. This is probably because Gd^3+^ occupies the Fe^2+^ cube [28]. The elements in the nanocluster were analyzed using energy-dispersive X-ray spectroscopy (EDS) mapping scans. As shown in Figure 1e, the nanoclusters are mainly composed of Fe and Gd, and Gd is evenly distributed in the nanoclusters. The Fe/Gd ratio was approximately 4/1, as determined by inductively coupled plasma–mass spectrometry (ICP–MS), which was the same ratio as that of the precursor before the reaction.

**Figure 2:**
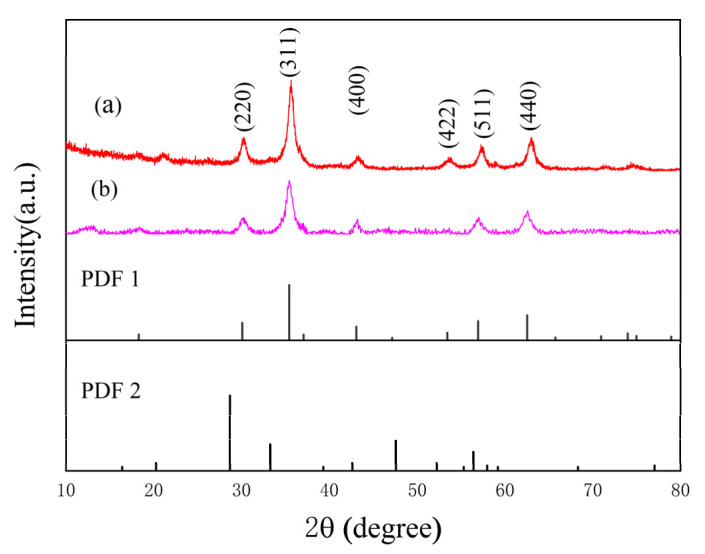
X-ray diffraction of (a) Fe_3_O_4_ nanoclusters, and (b) GdIO nanoclusters. JCPDS card (99-0073) for Fe_3_O_4_ (PDF 1) and (11-0604) for Gd_2_O_3_ (PDF 2).

Fe_3_O_4_ and GdIO were synthesized by the same method, and X-ray diffraction (XRD) was used to further analyze the phase structure of the nanoclusters (Figure 2). XRD analysis shows that the diffraction peaks of Fe_3_O_4_ and GdIO corresponded to the standard card of ferric oxide (JCPDS No. 99-0073). In addition, there were no other diffraction peaks in the GdIO sample, indicating that the sample is not a simple physical mixture of Gd_2_O_3_ and Fe_3_O_4_, and Gd^2+^ may occupy the tetrahedral or octahedral position of Fe_3_O_4_. Compared to Fe_3_O_4_, the diffraction peak of GdIO shifted slightly to the left, which is due to the larger radius of the Gd ion leading to an increase in crystal plane spacing [29], a result that is consistent with earlier outcomes.

**Figure 3:**
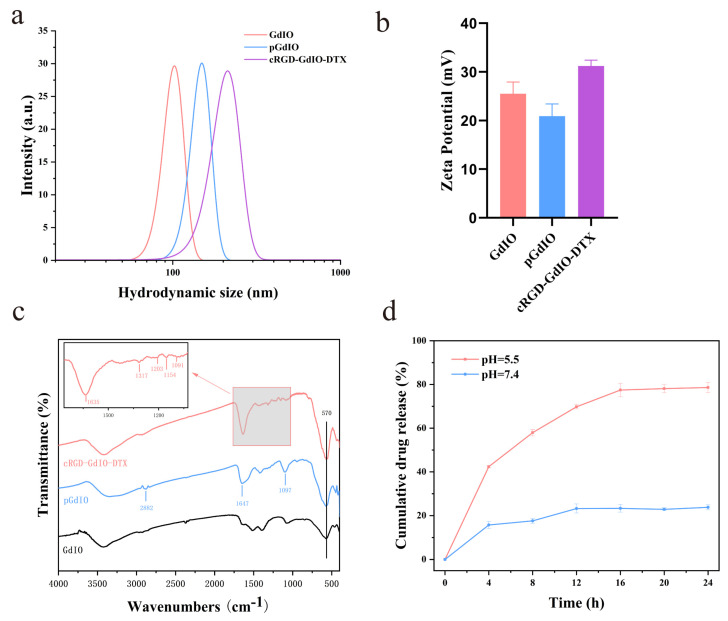
(**a**) hydrodynamic diameters, (**b**) zeta potentials, (**c**) FT-IR spectra of GdIO, pGdIO and cRGD−GdIO−DTX; (**d**) DTX in vitro release of cRGD−GdIO−DTX an different pH values (pH 5.5 and pH 7.4).

To achieve tumor targeting and therapeutic effects, a PEG_600_ diacid modification was made on the surface of the nanoclusters to allow cRGD and DTX binding. Subsequently, sample preparation was verified by different methods. As shown in Figure 3b, the GdIO nanoclusters have a high positive potential, but the zeta potential of PEG-GdIO (pGdIO) decreased to 20.93 mV due to the successful incorporation of PEG_600_ diacid with the COO- group. However, the zeta potential of cRGD-GdIO-DTX sharply increased again, which may be because many negative carboxyl groups were consumed in the process of cRGD, DTX and pGdIO conjugation. Moreover, cRGD and DTX provided positively charged amine and carbonyl groups, respectively. The hydrodynamic diameters of GdIO, pGdIO and cRGD-GdIO-DTX were measured in deionized water (Figure 3a). With continuous modification of the GdIO surface, the hydrodynamic diameter also increased, and the hydrodynamic diameters were 102, 149, and 212 nm. Additionally, the particle size measured by dynamic light scattering (DLS) was larger than that measured by TEM. This is most likely because of the hydration layer and small amount of aggregation that nanoclusters experience in aqueous environments [30]. Figure 3c shows the infrared spectra of GdIO, pGdIO, and cRGD-GdIO-DTX. The absorption peaks in all samples at 570 cm^−1^ are due to the stretching vibration of the Fe-O bond from the iron oxides. New absorption peaks appeared in pGdIO at 1097 and 2882 cm^−1^, which were C-O-C and C-H stretching vibrations attributed to PEG_600_ diacid, respectively [31,32], indicating the successful modification of PEG_600_ diacid. The vibration band of the carboxylic acid group of PEG is at about 1730 cm^−1^ [33]. When carboxylic acid interacts with metal ions, the carboxylic acid functional group is transformed into carboxylate, and the absorption peak moves towards the lower wavenumber of 1647 cm^−1^ [34]. In addition, the absorption peaks at 1154 and 1091 cm^−1^ in the cRGD-GdIO-DTX spectrum were attributed to the C-O stretching vibration of ester in DTX, and the absorption peak at 1317 cm^−1^ was attributed to the symmetric angular variable vibration of CH_3_. These results indicated the successful modification with the therapeutic group DTX. It is worth noting that DTX was reacted with the free carboxyl by an esterification reaction and only the C2 hydroxyl group could react through ester bonds because of the steric effect according to the reference [35]. The absorption peaks at 1203 cm^−1^ and 1635 cm^−1^ are attributed to C-N stretching vibration and amine groups variable angle vibration in cRGD, respectively, which indicates the successful modification of the targeted group cRGD. These results confirm the successful preparation of cRGD-GdIO-DTX.

### 2.2. Drug Loading and Release Behavior

The microenvironment of tumor cells is weakly acidic [36,37]. Therefore, the DTX that is connected to GdIO is released in response to pH, which helps kill tumor cells more efficiently. We calculated that the drug loading rate of DTX was 7.87% using UV–vis spectroscopy. Subsequently, we investigated the DTX release behavior from GdIO-DTX in neutral and weakly acidic phosphate buffers using UV–vis spectroscopy (230 nm). The DTX release curve is shown in Figure 3d. GdIO-DTX released only a small amount of DTX at pH = 7.4, while significantly more DTX was released in the weakly acidic environment at pH = 5.5. This typical pH-dependent release can decrease DTX release in a neutral environment, prolong drug circulation in the blood, and promote the release of DTX at the tumor site, thus reducing the negative side effects to normal tissues.

### 2.3. Magnetic Properties and Relaxivity of the Nanoclusters

**Figure 4:**
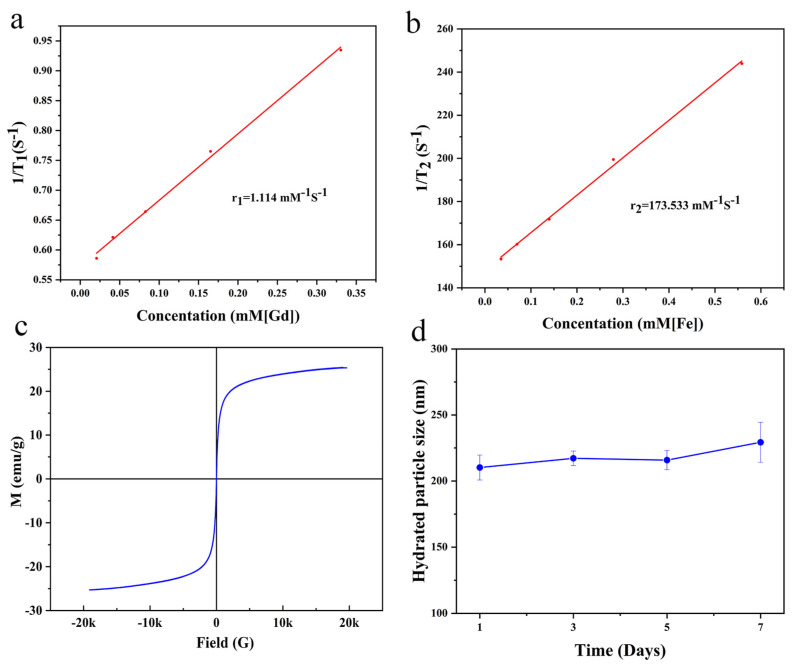
(**a**) T_1_ relaxation rate of pGdIO; (**b**) T_2_ relaxation rate of pGdIO; (**c**) Hysteresis loops of pGdIO at 300 K; (**d**) the size change of cRGD−GdIO−DTX after incubation in ddH_2_O for different periods.

The outstanding MRI performance of the nanoclusters was largely due to their magnetism. Therefore, a vibrating sample magnetometer (VSM) was used to examine the magnetic properties of GdIO. According to the field-dependent magnetization curves (Figure 4c), GdIO exhibited saturation magnetization with a value of approximately 25.4 emu/g at 300 K, indicating that it was capable of significantly reducing the T_2_ relaxation time of water molecules [38,39]. Additionally, there was no discernible coercive force or remanence at 300 K, demonstrating that GdIO exhibited exceptional superparamagnetism. Additionally, transverse and longitudinal relaxation rates play significant roles in determining how well CAs contrast on MRI [40]. A 0.5 T NMI 20 Analyst NMR instrument was then used to study the relaxivity of GdIO. The longitudinal (r1) and transverse (r2) relaxation rates of GdIO were 1.114 mM^−1^s^−1^ and 173.533 mM^−1^s^−1^, respectively (Figure 4a,b). These findings showed that GdIO has outstanding magnetic characteristics and is a good dual-mode MRI CAs candidate.

### 2.4. Biocompatibility and Biotoxicity

**Figure 5:**
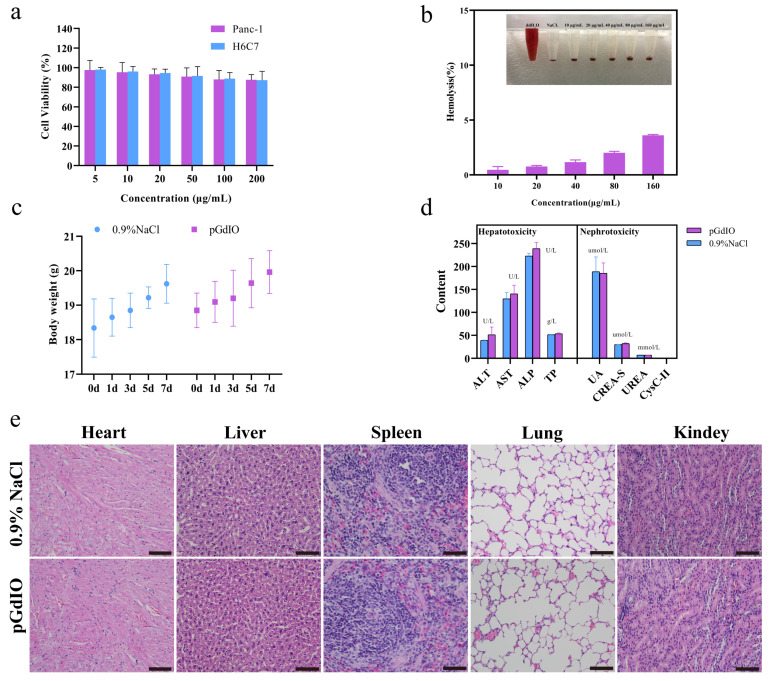
(**a**) Viability of Panc-1 cells and H6C7 cells upon treatment with pGdIO for 24 h; (**b**) hemolytic efficiency of pGdIO to human red blood cells; (**c**) weight changes of mice after treatment for a week, with data expressed as means ± SD (*n* = 5); (**d**) blood chemistry results for mice injected with pGdIO and 0.9% Nacl solution (*n* = 3); (**e**) H&E staining of organs dissected from mice upon treatment with 0.9% NaCl and pGdIO solution (scale: 50 μm).

To be useful for biomedical applications, these nanoclusters must have favorable biocompatibility, colloidal stability and lower biotoxicity. In this work, the colloidal stability of cRGD-GdIO-DTX was studied by determining the changes in the hydrodynamic size of cRGD-GdIO-DTX in deionized water. As shown in Figure 4d, there was no significant change in the hydrodynamic size of cRGD-GdIO-DTX in deionized water over one week, indicating that cRGD-GdIO-DTX has good colloidal stability. In addition, the release of free Gd^3+^ from the nanoclusters is an important factor to consider. As previously reported [41,42], the deposition of gadolinium ions in the body may lead to nephrogentic systemic fibrosis, which is very dangerous to the human body, especially in patients with acute renal failure. Based on this, we measured the leakage of the Gd element by ICP-OES analysis. The results show that the release of Gd is less than 2% after 1 day and 6 days of storage (Figure S1, Supplementary Materials), indicating that Gd has good chemical stability in nanoclusters [13]. The CCK-8 assay was used to investigate in vitro cytotoxicity. As shown in Figure 5a, the viabilities of Panc-1 and H6C7 cells were over 80% even after treatment with 200 mg/mL pGdIO, demonstrating that pGdIO had low cytotoxicity. The hemocompatibility of pGdIO was then evaluated using a hemolysis assay. As shown in Figure 5b, at all concentrations, the hemolysis rate was under 5%, satisfying the hemolysis requirement for biomaterials [43,44].

Healthy Balb/c mice were injected with pGdIO solution (5 mg/kg) or 0.9% NaCl solution. The aforementioned animals were then observed for seven days to assess the in vivo biotoxicity of the pGdIO nanoclusters. The body weights of the nude mice grew marginally in both the saline and pGdIO groups, as seen in Figure 5c. Additionally, blood tests revealed that the mice given pGdIO injections did not exhibit any overt signs of nephrotoxicity or hepatotoxicity (Figure 5d). The primary organs of the pGdIO-treated animals, such as the heart, liver, spleen, lung, and kidney, did not show any pathological alterations, according to the findings of the H&E staining experiment (Figure 5e). These findings point to pGdIO having good biocompatibility and the potential for extensive use.

### 2.5. Cellular Uptake of the Nanoclusters In Vitro

**Figure 6:**
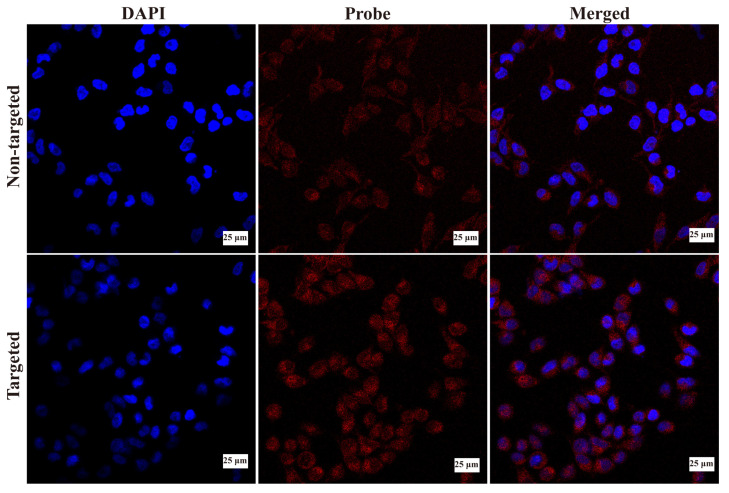
CLSM images of Panc-1 cells incubated with GdIO-DTX-Cy5.5 and cRGD-GdIO-DTX-Cy5.5, where red and blue represent Cy5.5 and DAPI fluorescence, respectively.

Effective tumor imaging and therapy depend heavily on the effective cellular uptake of MRI nanoagents. Therefore, an investigation of the cellular uptake of GdIO-DTX and cRGD-GdIO-DTX was undertaken. In this study, the cellular uptake of the nanoclusters was directly observed using confocal laser scanning microscopy (CLSM) with Cy5.5-labeled GdIO-DTX and cRGD-GdIO-DTX. As shown in Figure 6, strong red fluorescence was observed in Panc-1 cells after GdIO-DTX treatment, demonstrating that GdIO-DTX could efficiently enter cancer cells. In comparison to GdIO-DTX, the cells treated with cRGD-GdIO-DTX displayed more intense red fluorescence, indicating that cRGD-GdIO-DTX had superior cellular uptake. Then, Image-Pro Plus and GraphPad Prism 8 were used to obtain and analyze the integrated optical density (IOD) of each group of cells. The results also showed that the signal intensity of the targeted group was significantly higher than that of the non-targeted group (Figure S2). Integrin α_v_β_v_ can be particularly highly expressed by Panc-1 cells, and the cyclic RGD peptide can preferentially bind this integrin [45]. As a result, the targeting with cyclic RGD encourages Panc-1 cells to take up cRGD-GdIO-DTX.

### 2.6. Antitumor Effect of the Nanoclusters In Vitro

**Figure 7:**
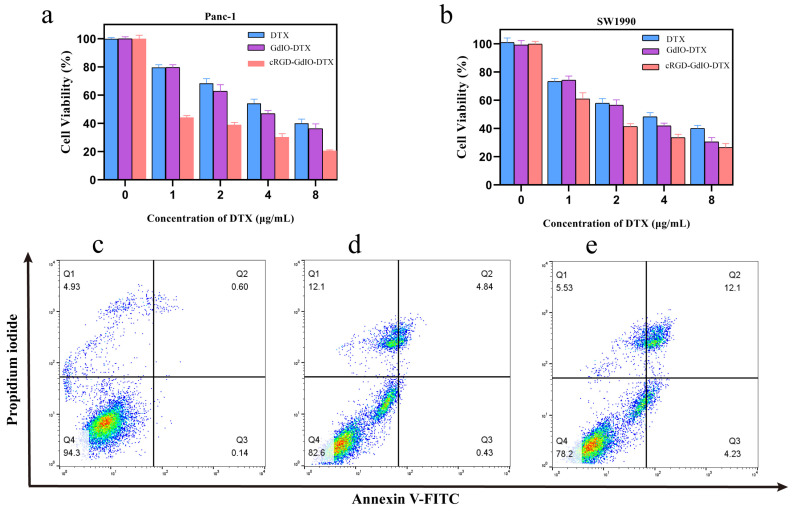
Relative viability of (**a**) Panc-1 and (**b**) SW1990 cells with free DTX, pGdIO-DTX and cRGD-GdIO-DTX at different DTX concentrations; (**c**–**e**) the apoptosis rates of Panc-1 cells co-cultured with PBS, GdIO-DTX and cRGD-GdIO-DTX nanoclusters for flow cytometry apoptosis data.

Intracellular cRGD-GdIO-DTX can release DTX. Then, it can impede cell division and arrest cells in G2/M phase, resulting in cancer cell death [46]. As shown in Figure 7a,b, the therapeutic effect of cRGD-GdIO-DTX was assessed using SW1990 and Panc-1 cells. Notably, all groups showed a considerable increase in Panc-1 and SW1990 cell mortality as the DTX concentration increased. More importantly, the mortality in the cRGD-GdIO-DTX group was considerably greater than that in the nontargeted GdIO-DTX group at equal doses of DTX, indicating that cRGD-GdIO-DTX has a better ability to inhibit tumor proliferation and could kill pancreatic cancer cells in a targeted way. Subsequently, an apoptosis experiment was performed, and the flow cytometry results are presented in Figure 7c–e. The apoptotic rates of Panc-1 cells treated with PBS, GdIO-DTX, and cRGD-GdIO-DTX were 0.74%, 5.27%, and 16.33%, respectively. Compared to GdIO-DTX, cRGD-GdIO-DTX induced more Panc-1 cell apoptosis. Accordingly, the successful conjugation between the targeting moiety cRGD and the nanoclusters enhanced nanocluster targeting and efficiently increased the apoptosis of pancreatic cancer cells, which reinforces the therapeutic effect of DTX.

### 2.7. Antitumor Effect of the Nanoclusters In Vivo

**Figure 8:**
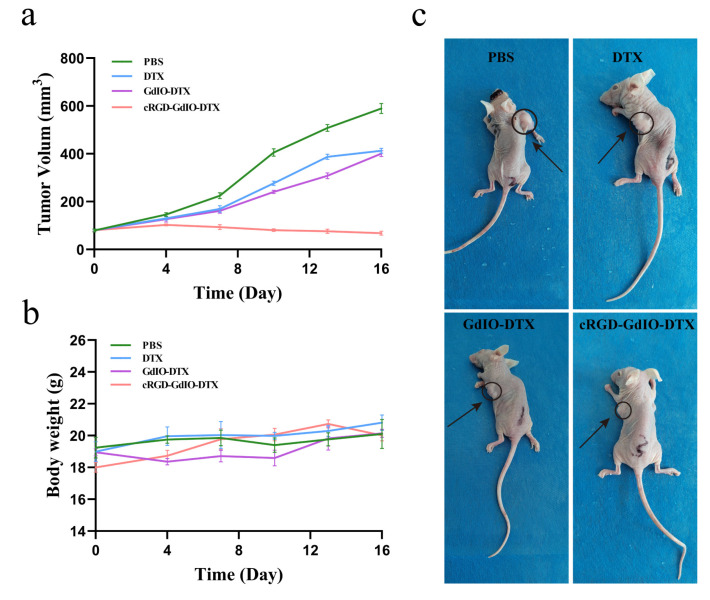
(**a**) Tumor volume growth trend of nude mice in different treatment groups; (**b**) body weight of nude mice in different treatment groups; and (**c**) tumor treatment effect graph.

The outstanding efficacy of cRGD-GdIO-DTX in vitro inspired us to further explore its antitumor ability in vivo. The tumor volumes of cancer-bearing mice were recorded after the intravenous injection of cRGD-GdIO-DTX at a dose of 2 mg/kg. As shown in Figure 8a, DTX and GdIO-DTX evidently restrained the tumor volume in comparison with the saline group. The targeting ligand accelerated the accumulation of cRGD-GdIO-DTX in the tumor site, which in turn strengthened the antitumor activity, which is why cRGD-GdIO-DTX demonstrated the strongest tumor inhibition. Moreover, as shown in Figure 8b, the nude mice in all groups maintained normal weight gain, and no significant abnormalities were observed.

**Figure 9:**
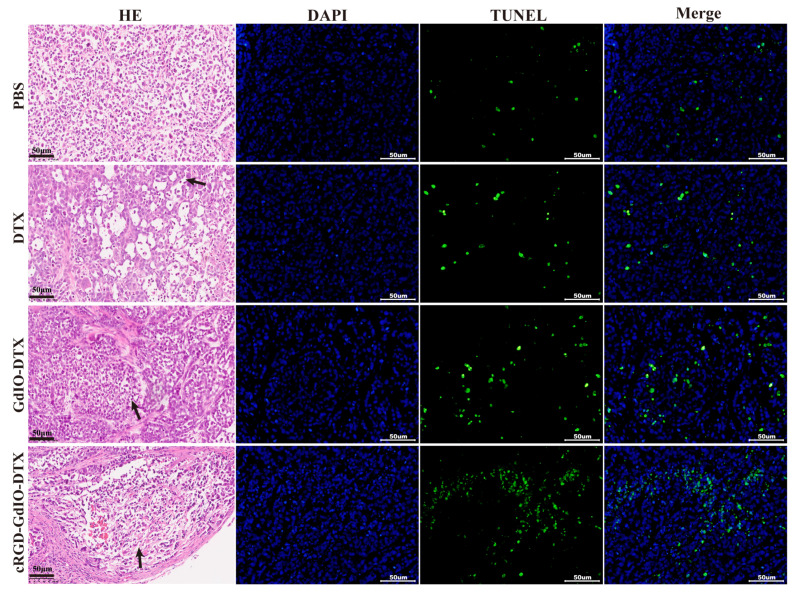
H&E staining and TUNEL assays of tumor tissues after treated with PBS, DTX, GdIO-DTX and cRGD-GdIO-DTX (The arrows show degeneration and necrosis of tumor cells).

At the end of treatment, some of the tumor tissues were cut into slices and analyzed further using H&E staining and a TUNEL assay. As shown in Figure 9, the nude mice treated with cRGD-GdIO-DTX had the fewest cancer cells but higher ratios of apoptosis and necrosis than the other treatment groups. All of the above results suggest that the binding of cRGD can help these nanoclusters target tumors more efficiently and produce more effective antitumor effects.

### 2.8. MR Imaging In Vivo

**Figure 10:**
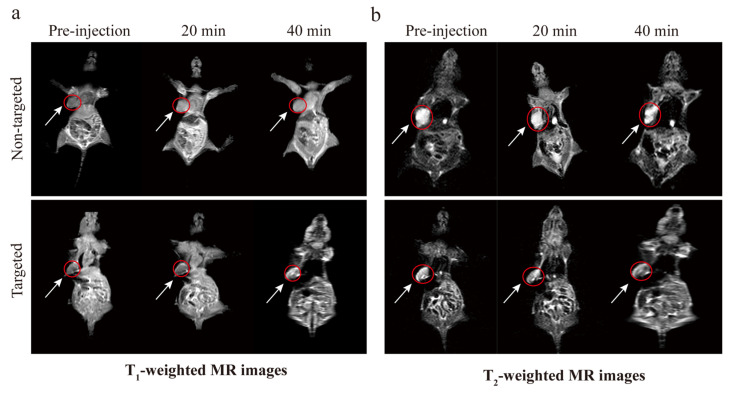
(**a**) T_1_-weighted and (**b**) T_2_-weighted MR images of nude mice at different time points post-injection of cRGD-GdIO and cRGD-GdIO-DTX.

As we mentioned earlier, GdIO exhibits outstanding magnetism, which motivated us to learn more about its MRI contrast capabilities. Using a 3.0 T MRI Scanner, we assessed the in vivo magnetic resonance imaging capabilities of cRGD-GdIO-DTX. Following injections of GdIO-DTX and cRGD-GdIO-DTX at a dose of 5 mg/kg, T_1_-weighted images (T_1_WIs) and T_2_-weighted images (T_2_WIs) of nude mice in the coronal plane were obtained at various intervals. As shown in Figure 10, the T_1_WIs of the tumor tissue gradually became brighter after injection of GdIO-DTX. Compared to GdIO-DTX, the T_1_WIs of the tumors from nude mice injected with cRGD-GdIO-DTX were evidently brighter, which could be attributed to the outstanding targeting ability of the nanocluster. Additionally, the T_2_WIs of the tumors from nude mice that had been treated with various nanoclusters darkened progressively, which was similar to the T_1_WI results. These findings showed that the systemic administration of cRGD-GdIO-DTX can hasten early tumor detection and allow precise treatment. It is therefore possible to draw the conclusion that cRGD-GdIO-DTX is a potential candidate with which accurate and effective cancer theranostics can be achieved.

## 3. Materials and Methods

### 3.1. Materials

The following reagents were purchased from Aladdin Co. (Shanghai, China) and used as received: Fe(acac)_3_ (98%), ethylene glycol (EG, 99%), Gd(acac)_3_ (99%), triethanolamine (TEA, 98%), diethylene glycol (DEG, 99%), dimethyl sulfoxide (DMSO), *N*,*N*′-dicyclohexylcarbodiimide (DCC), polyvinyl pyrrolidone (PVP), 4-dimethylaminopyridine (DMAP, 99%), and dichloromethane (98%). The cyclic RGD peptide and Cy5.5 were purchased from Zhongxiang Biotechnology Co, Ltd. (Xian, China). PEG_600_ diacid (COOH-PEG-COOH) and DTX were purchased from Macklin Co., Ltd. (Shanghai, China). The CCK-8 kit, DAPI staining solution, penicillin–streptomycin solution, and trypsin cell digestion solution were purchased from Beyotime (Shanghai, China). The Annexin V-FITC/PI apoptosis assay kit was purchased from KGI Bio (Jiangsu, China).

### 3.2. Synthesis of RGD-GdIO-DTX

#### 3.2.1. Synthesis of GdIO Nanoclusters

The GdIO nanoclusters were synthesized using Si’s method with minor modifications [38]. Briefly, 0.05 g of Gd(acac)_3_ was added to 10 mL of EG and 20 mL of DEG, and the mixture was stirred continuously at 80 °C for 40 min. To this mixture was added 1 g of PVP, and stirring continued for an additional 45 min. Then, 3 mL of TEA was added to the above solution, which was stirred for 2 h. Finally, Teflon-lined stainless steel autoclaves were filled with the prepared solution and maintained at 220 °C for 30 h. The black product was collected and repeatedly rinsed with ethanol and deionized water and centrifuged.

#### 3.2.2. Synthesis of pGdIO Nanoclusters

Forty milligrams of GdIO and 0.6 g of PEG_600_ diacid were dispersed in deionized water. After ultrasonic shock for 45 min, stirring was continued for 24 h. Afterward, PEG-modified GdIO was purified using a dialysis bag, and pGdIO was obtained by freeze-drying the purified suspension.

#### 3.2.3. Synthesis of cRGD-GdIO-DTX

Twenty milligrams of pGdIO was dissolved in 10 mL of methylene chloride, followed by the addition of 11.2 mg of DCC, 5.9 mg of DMAP, 13.4 mg of DTX, and 7 mg of cRGD. The concoction was stirred for 18 h in the dark. The products were then obtained by centrifugation. Finally, the unreacted reagents were removed using the deionized water repeated brush protocol.

### 3.3. Characterization

Transmission electron microscopy (TEM, FEI TF20, Waltham, MA, USA) was used to examine the morphology and structure of the samples. The size distribution and zeta potential of the nanoclusters were measured using a Malvern nanoparticle size analyzer (Nano-ZS90) instrument (Worcestershire, UK). The crystal structure of the sample was analyzed using X-ray diffraction (XRD, Bruker D8 Advance, Waltham, MA, USA). The interactions between the samples were analyzed using Fourier transform infrared (FT-IR) spectroscopy (FT-IR 6800 JASCO, Marseille, France) in the range of 450–4000 cm^−1^.

### 3.4. Drug Loading and Release Behavior

The loading rate of the drug was measured by UV–Vis spectroscopy. In summary, 1 mL of DMSO was used to dissolve the weighed drugs, and the UV–Vis spectra were recorded. The quantity of unencapsulated DTX was estimated by measuring the absorbance at 230 nm. Previously, a calibration curve for DTX in DMSO was created in the concentration range of 1–50 μg/mL. The following equations was used to compute drug loading (DLC) based on differences in the optical absorbance data and molar concentration [40].

$$\mathrm{DLC}\;\left(\%\right)=\frac{\mathrm{weight}\;\mathrm{of}\;\mathrm{the}\;\mathrm{drug}\;\mathrm{in}\;\mathrm{the}\;\mathrm{CSNPs}}{\mathrm{weight}\;\mathrm{of}\;\mathrm{the}\;\mathrm{CSNPs}}\times 100\%$$

The nanoclusters were dispersed in 4 mL of PBS buffer (pH 7.4 or 5.5) and transferred to dialysis bags, which were then placed on a magnetic stirrer at 37 °C. Then, samples from each solution were taken at 0, 4, 8, 12, 16, 20, and 24 h, and the absorption was measured at 230 nm to calculate the release of DTX.

### 3.5. Magnetic Properties and Relaxivity of the Nanoclusters

A vibration sample magnetometer (VSM, Lakeshore 7404, Westerville, OH, USA) was used to construct the hysteresis regression line of the sample to observe its magnetic properties.

The T_1_ and T_2_ relaxation rates at different Gd concentrations (0.02065, 0.04129, 0.08258, 0.16517, and 0.33033 mM) and Fe concentrations (0.03491, 0.06981, 0.13963, 0.27925, and 0.5585 mM) were measured by a 0.5 T NMI 20 Analyst NMR system (Niumag, Suzhou, China).

### 3.6. Biocompatibility and Biotoxicity

The stability of the nanoclusters was evaluated by observing the variation in hydrated particle size of the nanoclusters in deionized water over a 7-day period. Briefly, the probe was dissolved in deionized water, and the hydrated particle size was measured and recorded after 1, 3, 5, and 7 days.

Samples were dissolved in different aqueous media (deionized water, normal saline, and phosphate buffered saline (PBS)) with a concentration of 1 mg/mL before analysis. Gd content in the cRGD-GdIO-DTX was determined by a Leeman Prodigy inductively coupled plasma-optical emission spectrometer (ICPOES) (Mason, OH, USA). The samples were digested by aqua regia and diluted with water before measurements.

Biological toxicity was analyzed using CCK-8, H&E staining, hemolysis assays, and blood tests. Briefly, cells were inoculated into 96-well plates at a density of 6 × 10^3^ cells per well and then cultured in RPMI 1640 containing 1% Streptomyces penicillin and 10% fetal bovine serum at 37 °C under 5% CO_2_ for 24 h. Then, various concentrations of pGdIO nanoclusters (5, 10, 20, 50, 100, and 200 µg/mL) were added to the culture medium. After incubation for 24 h, CCK-8 solution (10 µL) was added to the wells of the 96-well plate for further incubation for 2 h. Finally, the absorbance of each well was measured at 490 nm using a tablet reader (SAFIRE2, TECAN, Mennedoff, Switzerland). The following formula was used to calculate cell viability.

$$\mathrm{cell}\;\mathrm{viability}\;\left(\%\right)=\frac{{\mathrm{OD}}_{\mathrm{Sample}\;}-{\;\mathrm{OD}}_{\mathrm{blank}}}{{\mathrm{OD}}_{\mathrm{Control}}-{\mathrm{OD}}_{\mathrm{blank}}}\times 100\%$$

Blood compatibility was evaluated according to the previously reported hemolysis test [46,47,48,49,50]. Red blood cells (RBCs) were first separated from the remaining blood by centrifugation at 2500 rpm for 6 min at 4 °C. The RBCs were then repeatedly washed and purified with normal saline. Then, 2 mL of pGdIO (10, 20, 40, 80, or 160 μg/mL) was dissolved in brine with 2 mL of diluted RBC suspension (4% *v*/*v*). The system was then centrifuged for 10 min at 8000 rpm after incubation for 4 h at 37 °C. By using UV–Vis spectroscopy, the absorbance of the supernatant at 576 nm was measured. The hemolysis rate formula is as follows.

$$\mathrm{Hemolysis}\;\left(\%\right)=\frac{A_{\mathrm{Sample}}-A_{\mathrm{Negative}\;\mathrm{Control}}}{A_{\mathrm{Positive}\;\mathrm{Control}}-A_{\mathrm{Negative}\;\mathrm{Control}}}\times 100\%$$

The in vivo toxicity of pGdIO was examined in female nude mice (18–20 g). Qiqihar Medical University’s Animal Ethics Committee (No. QMU-AECC-2022-120) gave its approval to all of the studies. Nude mice were injected with saline or pGdIO via the tail vein. The nude mice were euthanized 7 days later, and the main organs (heart, liver, spleen, lung, and kidney) were removed for histological examination.

### 3.7. Cellular Uptake of the Nanoclusters In Vitro

Cellular uptake of the nanoclusters was determined using a confocal laser microscope. Qiqihar Medical University’s Molecular Imaging Laboratory donated the Panc-1, H6C7 and SW1990 cells. Panc-1 cells were injected onto confocal culture dishes for 24 h of culture until they reached the logarithmic growth stage. Following cell adhesion, 50 µg/mL pGdIO or cRGD-GdIO was added for incubation. Then, the cells were rinsed with PBS to remove excess pGdIO and cRGD-GdIO that had not been taken up by the cells. The PBS-washed cells were then fixed with 4% paraformaldehyde for 20 min before being treated with DAPI for 10 min. Finally, the cellular uptake of the probe was observed by confocal microscopy.

### 3.8. Antitumor Effect of the Nanoclusters In Vitro

The in vitro therapeutic efficacy of nanoclusters carrying DTX was evaluated. Free DTX, GdIO-DTX, and cRGD-GdIO-DTX (DTX concentrations of 0, 1, 2, 4, and 8 μg/mL) were cultured with Panc-1 and SW1990 cells for 24 h, and the cell survival rate was determined colorimetrically by CCK-8 assays. The assay method and data processing were performed in the same manner as for the cytotoxicity assay.

A 6-well plate was initially filled with pancreatic cancer cells at a density of 2 × 10^5^/mL to evaluate the effect of cRGD-GdIO-DTX on apoptosis. After 12 h of culture, cRGD-GdIO-DTX, GdIO-DTX, and PBS (blank group) were added to each well, and incubation continued for 48 h. In this work, cell apoptosis was identified using flow cytometry.

### 3.9. Antitumor Effect of the Nanoclusters In Vivo

Nude mice with tumors were randomly assigned to one of four groups (*n* = 4) and given tail vein injections of 100 μL of PBS, DTX, GdIO-DTX, or cRGD-GdIO-DTX (2 mg/kg). The first dose was given on the first day of the experiment, followed by an additional dose every other day for a total of 15 days. Starting on day one, the mice were weighed on an electronic scale, and the tumor length and breadth were measured every other day with a Vernier caliper. Tumors were later removed, and all nude mice were sacrificed after the treatment cycle. The tumor tissues were then sectioned, embedded in paraffin, and preserved in a 4% paraformaldehyde solution. For histological observation, tumor staining and TUNEL assays were performed with H&E and optical microscopy. The formula shown below was used to calculate the tumor volume.

$$V=\frac{1}{2}{\mathrm{ab}}^{2}$$

### 3.10. MR Imaging In Vivo

For in vivo magnetic resonance imaging, 100 μL of cRGD-GdIO-DTX, and GdIO-DTX were injected into tumor-bearing nude mice via the caudal vein. Images were collected at 0, 20, and 40 min after injection with a 3.0 T magnetic resonance scanner. 

### 3.11. Statistical Analysis

Statistical data were analyzed using SPSS 22.0 software with a two-tailed Student’s *t* test. The standard deviations (SDs) are shown as error bars. Differences were considered statistically significant when *p* < 0.05.

## 4. Conclusions

In this study, we developed a system (cRGD-GdIO-DTX) for magnetic resonance bimodal imaging and targeted drug delivery. The colloidal stability, hemolysis assay, and toxicity experiments results suggested that cRGD-GdIO-DTX has good stability and biocompatibility. The in vitro cellular uptake experimental results showed that cRGD-GdIO-DTX had a good targeting effect on pancreatic cancer cells. In addition, the systemic delivery of cRGD-GdIO-DTX greatly improved the MRI contrast effect and restricted tumor development in a human pancreatic cancer xenograft model. Therefore, the nanoclusters investigated here can be employed for T_1_–T_2_ dual-modal MRI and tumor-targeted DTX delivery. However, our cell-based and animal-based explorations are not enough to represent medical applications, which require further in-depth studies to properly evaluate the efficacy of these nanoclusters.

## Data Availability

Data will be made available on request.

## Supplementary Materials

The following supporting information can be downloaded at: https://www.mdpi.com/article/10.3390/molecules28166134/s1, Figure S1. Gd contents (% of original) in the supernatants of cRGD-GdIO-DTX after process of storage (at 37 °C for 1 and 6 days, respectively) and centrifugation (12,000 rpm, 15 min). Figure S2. Integrated optical density (IOD) of Targeted and Non-targeted groups. Figure S3. Molecular structure of docetaxel (DTX).

## Sample Availability

Samples of the compounds are available from the author.
