# Supplementary material for: cRGD-Conjugated GdIO Nanoclusters for the Theranostics of Pancreatic Cancer through the Combination of T1–T2 Dual-Modal MRI and DTX Delivery
Source: Molecules. 2023 Aug 18;28(16):6134. doi: 10.3390/molecules28166134 (PMC10459307; doi:10.3390/molecules28166134)
Supplement: Supplementary file 1 [file molecules-28-06134-s001.zip › molecules-2481722-supplementary.pdf]

## Supplementary Materials

# cRGD-Conjugated GdIO Nanoclusters for the Theranostics of Pancreatic Cancer through the Combination of $T_1$-$T_2$ Dual-Modal MRI and DTX Delivery

Shengchao Wang [1], Guiqiang Qi [1], Zhichen Zhang [1], Qiangqiang Yin [1], Na Li [2], Zhongtao Li [1], Guangyue Shi [1], Haifeng Hu [3] and Liguo Hao [1,*]

1 Department of Molecular Imaging, School of Medical Technology, Qiqihar Medical University, Qiqihar 161006, China
2 Department of Imaging Medicine and Nuclear Medicine, School of Clinical Medicine, Jiamusi University, Jiamusi 154002, China
3 Medical Imaging Center, the Second Affiliated Hospital of Qiqihar Medical University, Qiqihar 161000, China

## List of contents:

**Page**

Figure S1.........................................................................................S2

Figure S2.........................................................................................S2

Figure S3.........................................................................................S3

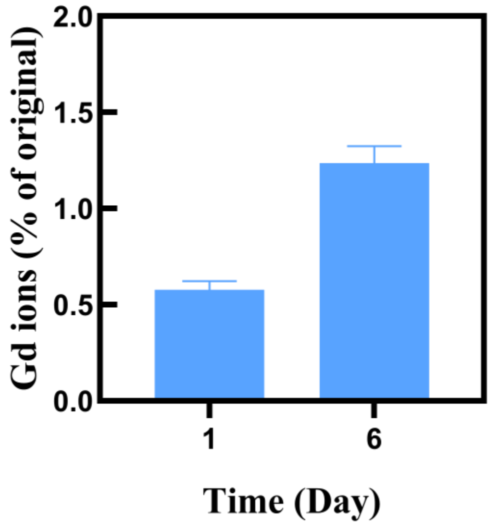


**Figure S1**. Gd contents (% of original) in the supernatants of cRGD-GdIO-DTX after process of storage (at 37℃ for 1 and 6 days, respectively) and centrifugation (12000 rpm, 15min).

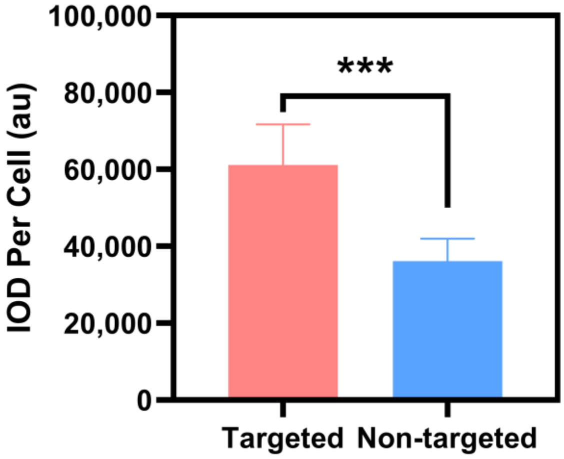


**Figure S2**. Integrated optical density (IOD) of Targeted and Non-targeted groups. *** $p < 0.001$

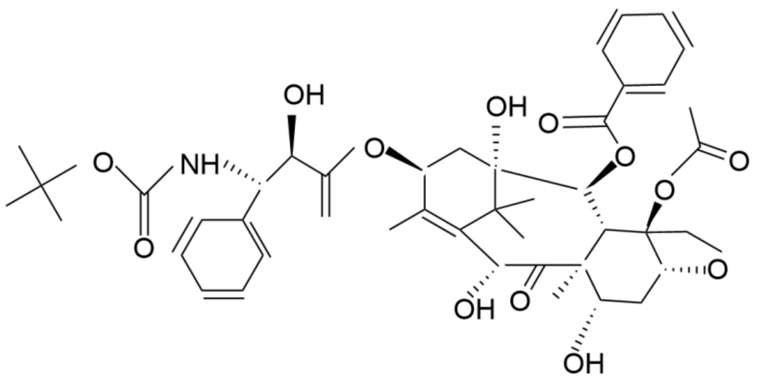


**Figure S3**. Molecular structure of docetaxel (DTX)
